# A pigmented calcifying cystic odontogenic tumor associated with compound odontoma: a case report and review of literature

**DOI:** 10.1186/1746-160X-3-35

**Published:** 2007-09-25

**Authors:** Phuu P Han, Hitoshi Nagatsuka, Chong H Siar, Hidetsugu Tsujigiwa, Mehmet Gunduz, Ryo Tamamura, Silvia S Borkosky, Naoki Katase, Noriyuki Nagai

**Affiliations:** 1 Department of Oral Pathology and Medicine, Graduate School of Medicine, Dentistry and Pharmaceutical Sciences, Okayama University, Okayama 700-8525, Japan; 2 Department of Oral Pathology, Oral Medicine and Periodontology, Faculty of Dentistry, University of Malaya, 50603 Kuala Lumpur, Malaysia

## Abstract

**Background:**

Pigmented intraosseous odontogenic lesions are rare with only 47 reported cases in the English literature. Among them, pigmented calcifying cystic odontogenic tumor, formerly known as calcifying odontogenic cyst, is the most common lesion with 20 reported cases.

**Methods:**

A case of pigmented calcifying cystic odontogenic tumor associated with odontoma occurring at the mandibular canine-premolar region of a young Japanese boy is presented with radiographic, and histological findings. Special staining, electron microscopic study and immunohistochemical staining were also done to characterize the pigmentation.

**Results:**

The pigments in the lesion were confirmed to be melanin by Masson-Fontana staining and by transmission electron microscopy. The presence of dendritic melanocytes within the lesion was also demonstrated by S-100 immunostaining.

**Conclusion:**

The present case report of pigmented calcifying cystic odontogenic tumor associated with odontoma features a comprehensive study on melanin and melanocytes, including histochemical, immunohistochemical and transmission electron microscopic findings.

## Background

Pigmented odontogenic lesions are rare, with only 47 cases reported in English literature since 1961 [Table 1]. Most of these pigmented lesions were found in racially pigmented patients. This is a case report of a pigmented calcifying cystic odontogenic tumor (CCOT) with special, ultrastructural and immunohistochemical findings together with a brief review of the English literature on pigmented odontogenic lesions especially pigmented CCOT.

**Table 1:** Reported cases of pigmented intraosseous odontogenic lesions^2–8, modified)^

**Diagnosis**	**No. of cases**	**Race of the patient**
Calcifying cystic odontogenic tumor	20	7 Japanese
		3 Black
		2 Indian*^one patient from 5^
		1 White
		1 Chinese*^5^
		1 Malay*^5^
		1 West Indian
		1 Hispanic
		3 ND
Keratocystic odontogenic tumor	8	5 Japanese
(Odontogenic Keratocyst)		1 Black, USA
		1 White
		1 West Indian
Adenomatoid odontogenic tumor	3	1 Japanese
		1 Black
		1 Mixed (White & Indian)
Ameloblastic fibro-odontoma	3	3 Japanese
Odontoma (Complex)	2	2 Japanese
Odonto-ameloblastoma	1	Japanese
Ameloblastic fibroma	1	Black
Odontogenic fibroma	1	ND
Ameloblastic fibrodentinoma	1	Japanese
Unclassified Tumor (?CEOT)	1	Black
Malignant ameloblastoma	1	Japanese*^6^
Ameloblastic carcinoma	1	White*^7^
Primary intraosseous carcinoma	1	Japanese*^8^
Dentigerous cyst	1	Japanese
Lateral periodontal cyst	1	Black, Israel
Botryoid odontogenic cyst	1	Black, South Africa

Total	47 cases	

* Additional cases found in the literature, ND Not described, Modified from the previous review due to the overlapping of a case

## Case presentation

A 15-year-old Japanese boy was referred to the Okayama University Hospital by the orthodontist for management of a mixed radiolucent and radiopaque lesion in the left mandibular canine-premolar region detected during routine radiographic examination. The lesion was asymptomatic and the patient's medical and family histories were non-contributory.

**Figure 1:**
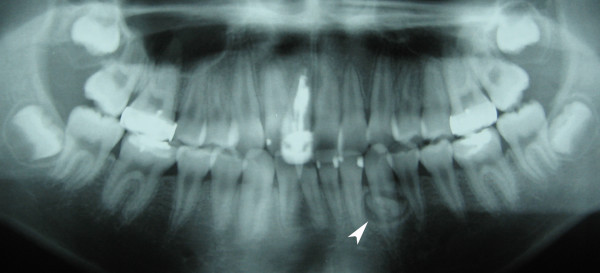
Panoramic radiograph demonstrated a well-defined radiolucent lesion with sclerotic border in left mandibular canine region (white arrowhead). Small, radio-opaque masses with comparable radio densities to that of the surrounding teeth were seen inside the radiolucent lesion.

On intraoral examination, there was no bony expansion and the overlying mucosa was also normal. Panoramic radiograph showed a well-defined unilocular radiolucent lesion with distinct sclerotic margin containing radiopaque masses [Fig. 1] and CT scan revealed that the lesion was lingual to the left mandibular canine. The lesion was small and the radiodensities of the included masses were comparable to that of the adjacent teeth. An initial diagnosis of odontoma was made, and surgical excision was performed under local anesthesia. Follicle-like tissue encapsulating three pieces of small calcified masses, was obtained from the surgical procedure.

**Figure 2:**
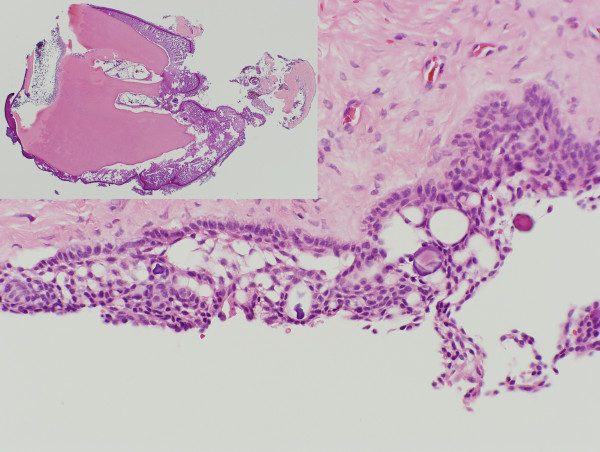
The epithelial lining (at the thickest portion) showed basal palisaded cuboidal cells and suprabasal stellate cells. Small focal calcifications within the epithelium can also be observed. (H&E, 20×). One of the small organoid denticles is shown in the inset.

Microscopic examination of the soft tissues revealed dental follicle-like loose connective tissue lined by a non-keratinized stratified epithelium of uneven thickness. The thickest portion of the epithelium showed basal cuboidal ameloblast-like cells and suprabasal stellate-like cells [Fig. 2]. Few round or drop-like calcifications were also observed focally within the epithelial lining.

Among the three calcified masses, two were small organoid denticles composed of enamel matrix, well-developed tubular dentin and pulp-like tissues with intervening loose connective tissues [Fig. 2, inset]. Ghost cells characterized by pale staining and ballooning cytoplasms and shadowy nuclei were seen within the odontogenic epithelium lining the enamel surface of the denticles as well as within calcified matrix. The other decalcified mass was an aggregate of ghost cells showing various degrees of dystrophic calcification. Focal and diffuse dark brown pigmentations, judged to be melanin granules, were recognized within the epithelial cells, connective tissue surrounding the denticles and also in the cytoplasms of ghost cells undergoing dystrophic calcification.

**Figure 3:**
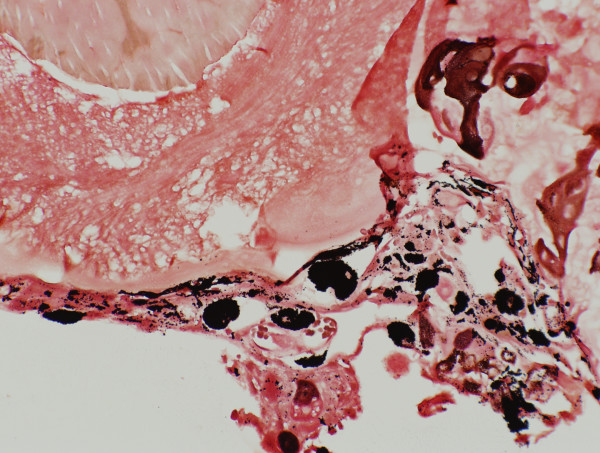
Odontogenic epithelium and ghost cells stained with Masson-Fontana for melanin pigmentation. Melanin pigments were detected within the epithelial cells, in the ghost cells and also lying freely within the extracellular connective tissue. (Masson Fontana, 40×).

**Figure 4:**
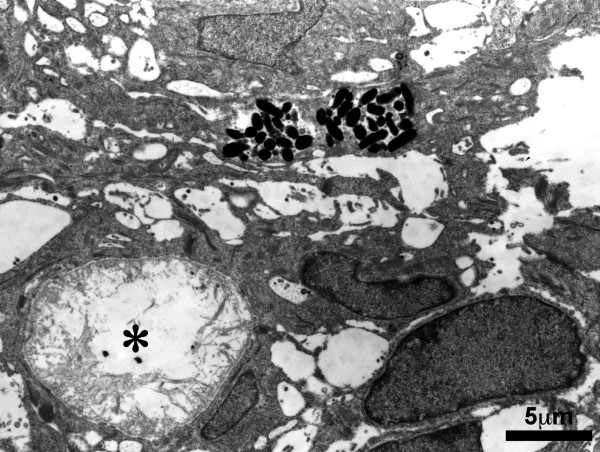
Transmission electron micrograph of the cyst lining epithelium. Melanosomes were seen within the epithelial cells. A pseudoglandular space lined by basement membrane was marked by asterisk (4,200×).

**Figure 5:**
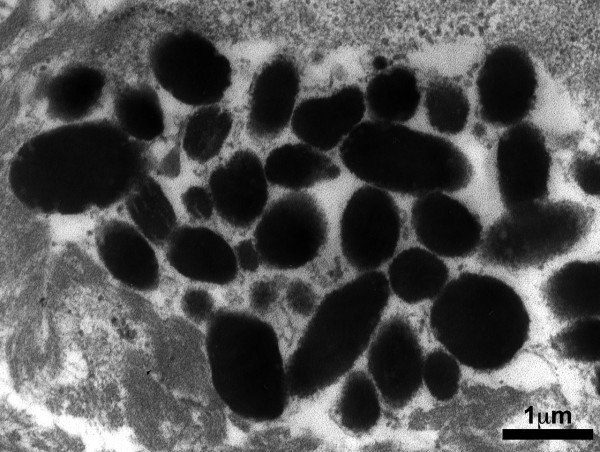
Irregular melanosomes with some inclusions could be observed with higher magnification under transmission electron microscope (24,000×).

**Figure 6:**
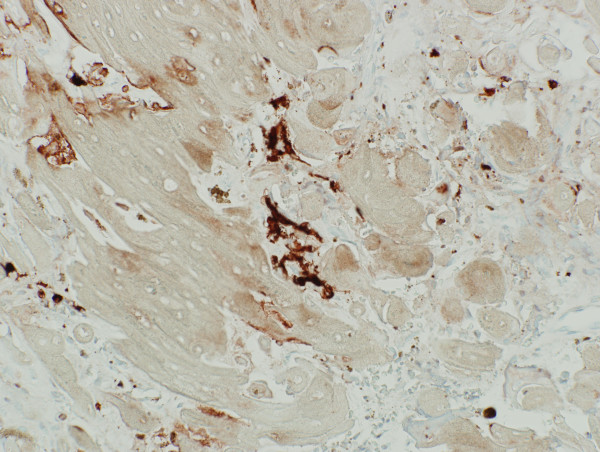
The presence of S-100 positive cells with cellular processes within the ghost cells aggregates (S100 immunostaining with AEC chromogen, 40×).

The melanin granules were confirmed by Masson-Fontana staining [Fig. 3] and melanosomes were detected by transmission electron microscopy (TEM) [Fig. 4, Fig. 5]. S-100 immunostaining revealed the presence of dendritic melanocytes within the ghost cell aggregates [Fig. 6].

## Discussion

A final diagnosis of pigmented CCOT associated with compound odontoma was undertaken due to the presence of well-developed denticles within the small cystic structure lined by typical odontogenic epithelium based on the criteria for the diagnosis of CCOT by the World Health Organization [1]. Reviews on pigmented odontogenic lesions [2-4] together with case reports from our literature search [5-8], revealed 47 cases. All these reported cases are summarized in Table I in order of abundance. The most commonly recognized pigmented odontogenic lesion is calcifying odontogenic cyst (COC), recently renamed as CCOT [1] with 20 reported cases [Table 2].

**Table 2:** Reported cases of pigmented calcifying odontogenic cyst in the literature^2, modified)^

**Author and Year**	**Age**	**Sex**	**Site**	**Race/Nationality**	**Odontoma**
Lurie HI(1961)*	23	F	Max	Bantu	Complex
Gorlin et al.(1964)*	16	M	Max	Unknown	
Duckworth & Seward(1965)	24	F	Max	Negro	
Abrams & Howell(1968)*	21	F	Max	Caucasian	
Chandi & Simon(1970)*	27	M	Man	From India	
Sauk(1972)	64	M	Man	Unknown	
Petri and Stump(1976)*	11	F	Max	Negro	
Saito et al.(1982)	13	M	Man	Japanese	
	9	F	Man	Japanese	Compound
	35	F	Max	Japanese	
Nagao et al.(1982)	13	F	Man	Japanese	Complex
Somes(1982)*	15	F	Man	West Indian	
Schwimmer et al.(1983)	13	M	Man	Hispanic	
Takeda et al.(1985b)*	21	M	Max	Japanese	
Keszler & Guglielmotti(1987)	15	F	Max	Unknown	Composite
Siar & Ng (1987)*	16	F	Max	Chinese	Compound
	31	M	Max	Indian	
	68	F	Man	Malay	
Takeda et al.(1990)	17	M	Man	Japanese	
	11	F	Man	Japanese	

* Studies with special staining to melanin pigmentation.

Among those pigmented CCOTs, 5 were associated with odontomas, 2 compound type and 3 complex or composite odontomas. All cases were intraosseous lesions and 10 out of 20 cases included special staining for melanin but only one with TEM examination. The present case report of pigmented CCOT associated with odontoma (CCOTaO) features a comprehensive study on melanin and melanocytes with special and immunohistochemical staining together with identification of melanosomes by TEM.

The exact etiology of melanin pigmentation in intraosseous odontogenic lesions is unknown, leaving room for speculation. As described in previous reports, most of the patients were Blacks and Asians, thereby implicating racial pigmentation to be an important factor [2,4]. Melanocytes, normally present in the oral mucosa, are also found in the dental lamina or tooth bud of the fetuses more commonly in pigmented race [9]. Odontogenesis is a complex process resulting from the reciprocal and close interactions between oral epithelium and cranial neural crest-derived ectomesenchyme [10]. It might not be surprising that melanocytes, which are also of neural crest in origin, may be present in dental lamina and odontogenic lesions. Another possibility is that a few proportion of lesional odontogenic tissue could have potential for neuroectodermal differentiation under certain circumstances. It is also rational to speculate that the quantity of melanocytes and the conditions or predisposing factors activating them for melanin production might be associated with racial pigmentation. Further studies are necessary to prove or refute these possibilities.

Although the 2005 WHO classification includes CCOT as a benign odontogenic neoplasm [1], CCOT features heterogenous histologic spectrum ranging from cystic to solid structure, and exhibits a variety of clinico-pathologic and behavioral characters [11,12]. Because of the diverse clinico-histologic features and the various neoplastic potential, there have been disagreements on the terminology as well as whether to classify CCOTs as a cyst or a neoplasm. Moreover, CCOT is frequently associated with other lesions such as odontoma, ameloblastoma and ameloblastic fibroma, and the most common of these is the CCOTaO [11,12]. The prevalence of CCOTaO was reported in 17.4% of 92 cases by Hong [11], 23.8% of 21 cases by Li TJ [12] and 35% of 215 cases by Buchner [13]. A concensus still lacks on the classification of CCOTaO either as a separate type of CCOT, i.e., combined odontogenic lesion with some proliferative potential [14,15] or as a sub-type of non-proliferative simple unicystic type of CCOT. Hirshberg analyzed 52 cases of CCOTaO and proposed CCOTaO to be regarded as a separate entity and suggested the term "odontocalcifying odontogenic cyst", due to the unique histologic features and its female predilection with the predominant distribution pattern to the maxilla [14]. However, it is generally accepted that CCOTaO occurs in a significantly younger age group compared to other types [11,14,17].

Pigmented compound odontoma was considered as a differential diagnosis because of the presence of ghost cells with subsequent calcification and that the occurrence of odontogenic epithelium is not rare in odontomas [18-20]. Combination lesions should also be anticipated because the odontogenic epithelium in different areas might undergo variable degrees of differentiation and degeneration [21]. In reality, the differentiation of CCOTaO from odontoma is difficult and subjective for some cases. There seems to be a mere difference in clinical behavior and growth potential between these two lesions and the controversy is merely of academic interest. The treatment for both lesions is conservative enucleation and the recurrence is very rare.

## Conclusion

This is a case of pigmented CCOTaO with comprehensive studies on melanin pigments that will add to the rare literature of pigmented odontogenic lesions. All the reported cases of pigmented intraosseous odontogenic lesions in the English literature since 1961, especially pigmented CCOT, were reviewed and summarized for academic purpose.

## Abbreviations

Calcifying cystic odontogenic tumor (CCOT), Calcifying cystic odontogenic tumor associated with odontoma (CCOTaO), Calcifying odontgenic cyst (COC)

## Competing interests

The author(s) declare that they have no competing interests.

## Authors' contributions

PPH, HN, CHS carried out the case study, discussed and reviewed the literature and prepared the manuscript. MG, RT and NN participated in the discussion and review process and also in critical revision of the manuscript. SB and KN helped in data collection and reviewing of the literature. All the authors have read and approved the manuscript.
